# Photoluminescence Study of the Interface Fluctuation Effect for InGaAs/InAlAs/InP Single Quantum Well with Different Thickness

**DOI:** 10.1186/s11671-017-1998-8

**Published:** 2017-03-29

**Authors:** Ying Wang, Xinzhi Sheng, Qinglin Guo, Xiaoli Li, Shufang Wang, Guangsheng Fu, Yuriy I. Mazur, Yurii Maidaniuk, Morgan E. Ware, Gregory J. Salamo, Baolai Liang, Diana L. Huffaker

**Affiliations:** 1 School of Science, Beijing Jiaotong University, Beijing, 100044 People’s Republic of China; 2 College of Physics Science and Technology, Hebei University, Baoding, 071002 People’s Republic of China; 3 Institute for Nanoscience and Engineering, University of Arkansas, Fayetteville, AR 72701 USA; 4 California NanoSystem Institute, University of California, Los Angeles, CA 90095 USA

**Keywords:** Photoluminescence, Quantum well, Interface, Nanostructures, Semiconductor

## Abstract

Photoluminescence (PL) is investigated as a function of the excitation intensity and temperature for lattice-matched InGaAs/InAlAs quantum well (QW) structures with well thicknesses of 7 and 15 nm, respectively. At low temperature, interface fluctuations result in the 7-nm QW PL exhibiting a blueshift of 15 meV, a narrowing of the linewidth (full width at half maximum, FWHM) from 20.3 to 10 meV, and a clear transition of the spectral profile with the laser excitation intensity increasing four orders in magnitude. The 7-nm QW PL also has a larger blueshift and FWHM variation than the 15-nm QW as the temperature increases from 10 to ~50 K. Finally, simulations of this system which correlate with the experimental observations indicate that a thin QW must be more affected by interface fluctuations and their resulting potential fluctuations than a thick QW. This work provides useful information on guiding the growth to achieve optimized InGaAs/InAlAs QWs for applications with different QW thicknesses.

## Background

In the past three decades, In(Ga)As/InAlAs quantum heterostructures grown on the InP surface have been extensively studied both experimentally and theoretically [1–5]. The InGaAs/InAlAs/InP quantum well (QW) heterostructures, including single quantum wells, multiple quantum wells, and superlattices, have special advantages for applications in long-wavelength optical communications as well as high-speed electronic and photonic devices [6–10]. First, the band structure and band offset of the InGaAs/InAlAs QW heterostructures can be flexibly tailored for different application purposes by varying either the composition or the thickness of the InGaAs active layer and the InAlAs barrier layer accordingly [11, 12]. Second, the wavelength of InGaAs/InAlAs QW inter-band transitions can cover the standard 1.31- and 1.55-μm optical communication wavelengths [13]. Third, the large conduction-band offset of the InGaAs/InAlAs system enables a strong electron confinement and subsequently a high-temperature stability and a high-speed modulation capability for InGaAs/InAlAs devices [14, 15]. This makes these structures very attractive and suitable for quantum cascade lasers, inter-subband detectors, and devices based on nonlinear optical properties [16–18]. In addition, the small electron effective mass, the small band gap, and the high electron mobility of InGaAs alloys are important for the development of high electron mobility transistors, high-speed detectors, modulators, and THz emitters [19–22].

Although InGaAs/InAlAs QW heterostructures possess special advantages for applications in long-wavelength and high-speed optoelectronic devices, they are very sensitive to alloy and interface fluctuations which can strongly affect the optical properties and performance of any resulting devices [23–25]. It is very necessary to acquire more detailed experimental data in order to precisely control the optical properties of the InGaAs/InAlAs QW heterostructures. In this letter, we carefully study the InGaAs/InAlAs interface effects by comparing the optical properties of two InGaAs/InAlAs/InP QWs with thicknesses of 7 and 15 nm. Interface fluctuation effect is evaluated by the evolution of the excitation intensity-dependent as well as temperature-dependent photoluminescence (PL) spectra. Finally, modeling of the QW band structure with varied thickness and alloy composition is presented to reveal the mechanisms underlying the PL observations. The modeling combined with PL measurements provides very useful information on guiding the growth to achieve optimized InGaAs/InAlAs QWs for applications with different QW thicknesses.

## Methods

Two QW samples were grown by molecular beam epitaxy (MBE) on the same day on two separate quarter pieces of the same 2-in semi-insulating InP(100) substrate at a growth temperature of 510 °C. The sample consisted of a lattice-matched In_0.53_Ga_0.47_As single quantum well (SQW) with nominal well widths of 7 or 15 nm. The InGaAs QW is sandwiched between a 70-nm In_0.52_Al_0.48_As barrier on the top and an 80-nm In_0.52_Al_0.48_As buffer layer underneath. In order to protect the top InAlAs layer from oxidation, a 3-nm lattice-matched InGaAs layer was grown as a final cap on top of the samples. For reference, one 300-nm InGaAs sample and one 300-nm InAlAs sample were also grown to calibrate the ternary alloy composition and lattice-match condition.

After growth, all samples showed mirror-like surface, and we did not observe haziness or cross-hatching under a Normaski microscope. Then, the QW samples were characterized by XRD and PL. For PL measurements, the samples were mounted in a closed-cycle cryostat (Janis CCS-150) with temperature variable from 10 to 300 K and excited by a 532-nm continuous-wave (CW) laser. The PL signal was detected by a liquid nitrogen-cooled CCD detector array (Princeton Instruments PyLoN: 1024-1.7) attached to a 50-cm focal-length spectrometer (Acton 2500).

## Results and Discussion

**Fig. 1:**
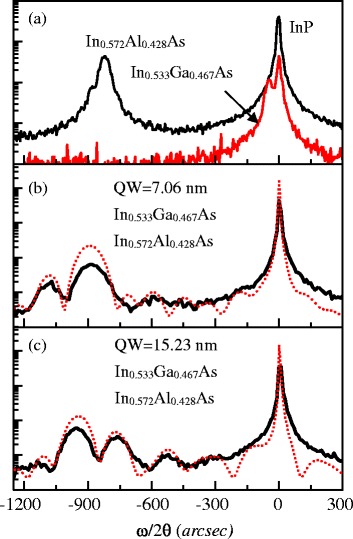
The X-ray diffraction results for **a** the bulk InAlAs and InGaAs reference samples, **b** the 7-nm InGaAs/InAlAs QW, and **c** the 15-nm InGaAs/InAlAs QW. The *solid curve* is the experimental data and the *dash curve* is the simulation results

The XRD *ω*/2*θ* (004) scan was measured for both QW samples and the two reference samples. As shown in Fig. 1a, the compositions were calibrated to be In_0.533_Ga_0.467_As for the QW active layer and to be In_0.572_Al_0.428_As for the barrier. Additionally, the QW thickness obtained from the simulation of XRD curves in Fig. 1b, c are 7.06 and 15.23 nm, respectively. The XRD measurements also show a very narrow (FWHM ≅ 20 arcsec) and strong peak from the InP substrate for all samples, which together with the epi-layer peaks indicate the good quality of InGaAs QW and InAlAs barrier layers.

**Fig. 2:**
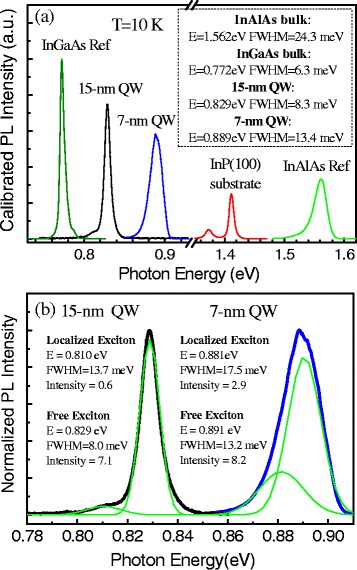
**a** The PL spectrum measured at 10 K with a laser intensity of 0.1 W/cm^2^ for the 7-nm QW, the 15-nm QW, the bulk InGaAs, and InAlAs reference samples; **b** the two-peak Gaussian fitting analysis for the PL spectrum of the 7-nm QW and the 15-nm QW to show the localized exciton emission and free exciton emission

Photoluminescence spectra for the two QWs, the InGaAs, and the InAlAs reference samples, as well as an InP(100) substrate measured at 10 K with a laser intensity of 0.1 W/cm^2^, are plotted in Fig. 2a for comparison. It can be seen that the QW emission shifts from 0.829 eV to a higher energy of 0.889 eV as the thickness decreases from 15 to 7 nm. This is as expected for a confined InGaAs/InAlAs QW system and allows the discrete energy level to be flexibly tailored in a broad range by only varying the well thickness. Meanwhile, we did not observe PL emission from the InAlAs barrier materials for either QW sample, indicating fast carrier relaxation into the QW from the InAlAs barrier layer due to the large conduction-band offset of the InGaAs/InAlAs QW system. The PL peak from the InGaAs reference sample is observed at 0.772 eV, which has a blueshift of ~31 meV from the well-recognized InGaAs bandgap (~0.803 eV) at 10 K [26]. It is likely due to the small lattice mismatch and the residual strain in the InGaAs epi-layer. In particular, the InGaAs reference sample shows a very narrow (FWHM ~6 meV) PL peak, indicating stable MBE growth conditions and very uniform composition distribution for the InGaAs ternary alloy epi-layer.

The QW PL spectra are analyzed more closely in Fig. 2b. Both samples exhibit anisotropic spectral profile. The spectra are fitted well with a double Gaussian profile. The stronger, higher energy peak in each sample is attributed to the free exciton transition (E1-H1) in the QW from the ground state of the electrons (E1) to the ground state of the holes (H1). The smaller peaks in each sample are attributed to localized excitons which exhibit slightly lower energies due to potential modulations inside the InGaAs and InAlAs alloys or at the InGaAs/InAlAs interface [25, 27, 28].

Both the imperfect hetero-interface and the composition modulations in ternary alloys that constitute the InGaAs QW and InAlAs barriers would lead to fluctuations in the confinement potential and may trap excitons in the localized potential minimum [28–30]. If the localized excitons are due to the composition modulations inside the InGaAs or InAlAs ternary alloys, considering the identical growth condition, the localized potential minimum and localized excitons should give similar PL performance for both QW samples. However, on the contrary, we observe that the localized exciton emission weights larger for the 7-nm QW. Its integrated PL intensity ratio (*R* = 0.35) between the localized exciton peak to the free exciton peak is larger than that of the 15-nm QW (*R* = 0.08). Therefore, we believe that the localized excitons are mainly the result of imperfect interfaces. An increase of the potential fluctuation due to the interface roughness has been observed as the well thickness decreases. At the same time, with the reduction of the QW thickness, a deeper penetration of the carrier wave functions occurs into the InAlAs barrier, presenting larger compositional disorder through the interface [28]. Additionally, the 7-nm QW has a broader linewidth (FWHM = 17.5 meV) than that of 15-nm QWs (FWHM = 13.7 meV) on the localized exciton peak, which also indicates that the 7-nm QW is affected more by the interface roughness fluctuations and compositional disorder.

**Fig. 3:**
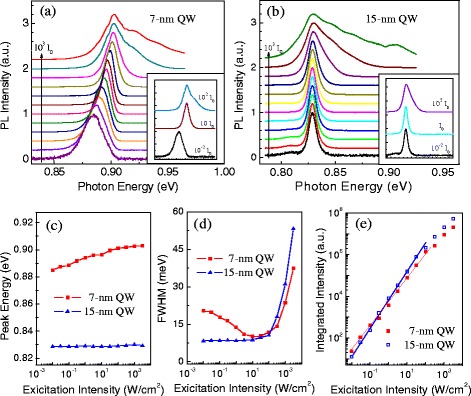
PL measured as a function of the excitation intensity from 10^−*2*^ *I*_0_ to 10^3^ *I*_0_ (*I*_0_ = 1 W/cm^2^). **a** PL spectra for the 7-nm QW. **b** PL spectra for the 15-nm QW. **c** PL peak energy as a function of excitation intensity. **d** FWHM of PL spectra as a function of excitation intensity. **e** Integrated PL intensity of the QW as a function of the excitation intensity. *Insets* in **a** and **b** are low, mid, and high excitation intensity examples of PL spectra to show the profile transformation with the increasing excitation intensity

We then study the two QWs carefully via excitation-dependent PL measurements at low temperature (*T* = 10 K). Figure 3a,b present PL spectra of the two QW samples, respectively. For convenience, each spectrum is normalized according to the maximum PL intensity and shifted up. The PL peak energy, FWHM, and integrated intensity of both QW samples are extracted and plotted in Fig. 3c–e as a function of the excitation intensity. Of particular importance here is that the PL from the 7-nm QW exhibits a large shift of 15 meV to higher energy as the laser intensity increases from 0.01 to 100 W/cm^2^. Then, its PL peak energy remains relatively stable for laser intensities between 100 and 3000 W/cm^2^. In comparison, PL peak energy of the 15-nm QW is stabilized at 0.829 eV for five orders of magnitude in laser intensity increasing from 0.01 to 3000 W/cm^2^, as shown in Fig. 3c.

The profile of the PL spectra from the 7-nm QW also undergoes a transformation with increased excitation intensity, as indicated by Fig. 3a and its inset. Under a weak excitation of 0.01 W/cm^2^, it starts from an asymmetric shape with the free exciton peak at the higher-energy side and the localized exciton peak at the lower-energy side. As the excitation increases from 0.01 to 10 W/cm^2^, the spectrum gradually becomes symmetric, and finally, a perfect Gaussian peak is visible. At the same time, the FWHM narrows from 20.3 to 10.0 meV, as shown in Fig. 3d. After that, the PL spectra become asymmetric again but with a shoulder on the higher-energy side. These features can be explained very well by the filling effect of the localized exciton at low excitation and a free exciton energy state at high excitation intensity [25, 28]. At very low excitation powers, the lowest localized excitons are preferentially populated, so a lower energy shoulder is clearly observed to correlate with the localized exciton emission. Then, they are filled with increased excitation intensity and quickly get saturated because the state density of localized excitons is much lower in comparison with that of the free excitons. After the localized excitons become saturated, the free exciton emission remains and increases with increasing excitation intensity. This finally becomes the dominant PL signal, exhibiting a narrow peak with symmetric Gaussian profile. However, with further increased excitation intensity, the PL again becomes asymmetric, broadening to higher energies due to excited states of the QW. In contrast, the PL spectra of the 15-nm QW also starts with an asymmetric shape but less localized exciton emission. It turns into a symmetric profile at 0.3 W/cm^2^, which is a much smaller excitation intensity than that for the 7-nm QW. Furthermore, the FWHM changes very little until the laser intensity finally exceeds 10 W/cm^2^, and the excited states of the QW become evident, broadening the peak to higher energy.

The excitation intensity-dependent PL measurements indicate that the narrower the QW, the greater the effect of localized excitons are on the QW optical properties. Figure 3a, b indicate the coexistence of the free exciton and localized exciton for both QWs, although the free exciton emission could be much stronger than the localized excitons. For the 7-nm QW, the initial blueshift of the PL peak energy with increasing laser intensity is a result of the gradual filling of the localized exciton states caused by interface imperfections. The variation of the FWHM also confirms the change from the very broad spectra of the localized excitons to the narrow-band spectra of the free excitons, as shown in Fig. 3d. For the 15-nm QW, the density of the free exciton levels in the QW is very high. The localized exciton states are much weaker than the free excitons, and the emission from localized excitons contributes only slightly to the PL emission. Therefore, it is hard to observe the filling effect of the localized exciton levels and the related large blueshift and narrowing of the PL peak.

We also see in Fig. 3e that the integrated PL intensity of the 7-nm QW is slightly stronger than that of the 15-nm QW under weak excitation, followed by a crossover when the laser intensity increases above ~1 W/cm^2^. It reveals that the PL intensity is not simply proportional to the QW volume in the experiments. This may be understood by considering that in both QWs, there are two general channels for relaxation of the excitation, the localized state and the free state of the exciton. Using a 532-nm CW laser for excitation, the photo-induced carriers are mainly generated inside the InAlAs barrier layer instead of directly inside the InGaAs QW. If we assume that under the same excitation intensity, the same amount of carriers are generated in the InAlAs barrier layer and then diffuse into the well in both samples, we would simply assume that more carriers would be captured by the 15-nm QW than the 7-nm QW due to the larger size, deeper ground state, and therefore larger captured cross-section. However, it is apparent from Fig. 3a, b that at low power, there are more carriers in the localized exciton states in the 7-nm QW and more carriers in the free exciton state in the 15-nm QW. Due to the larger oscillator strength of the localized exciton (3-dimensional confinement) compared to the free exciton (1-dimensional confinement) [31–33], the emission from the localized exciton is stronger than that from the free excitons, i.e., the 7-nm QW has stronger PL than the 15-nm QW under weak excitation condition. But, at much higher excitation intensities, the 7-nm QW is predominantly saturated of emissions from both localized exciton and free exciton states; the 15-nm QW, as expected, has stronger PL than the 7-nm QW. Therefore, we observe a crossover of the integrated PL intensity when the laser intensity increases to a certain level.

**Fig. 4:**
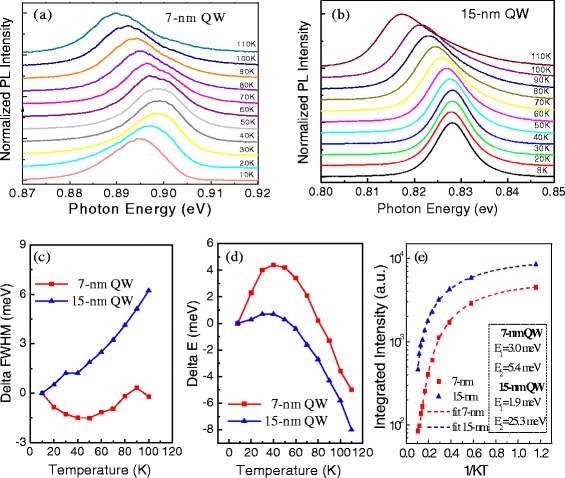
PL spectra as a function of temperature for **a** 7-nm QW sample and **b** 15-nm QW sample. **c** The PL FWHM, **d** PL peak energy shift, and **e** integrated PL intensity vary as a function of temperature for the 7-nm QW and the 15-nm QW measured at the laser excitation intensity of *I*_0_ = 1 W/cm^2^

Figure 4a, b show the results of temperature-dependent PL spectra for the two QW samples for which the results appear to be very different for each sample. First, in Fig. 4c, we can see that the FWHM of the 15-nm QW increases monotonically, while the FWHM of the 7-nm QW decreases first and then increases as the temperature increases from 10 to 110 K. Second, as the temperature increases, in Fig. 4d, both samples initially exhibit a shift of the PL peak to higher energy, up to a maximum shift at ~50 K. However, the maximum blueshift is 4.5 meV for the 7-nm QW and only 0.7 meV for the 15-nm QW. As the temperature increases higher from 50 to 110 K, the PL peaks of both QW samples shift to lower energies, although it can be seen that the redshift is faster for the 7-nm QW than the 15-nm QW.

Similar anomalous behavior of the PL peak energies with increasing temperature have been found in other InGaAs/InAlAs QWs in the lower temperature range [34, 35]. It has been explained by the presence of a band tail in the density of state (DOS) due to potential fluctuations resulting from interfaces in the QWs. When the localized excitons are photo-generated in the QWs with potential fluctuations, some of these excitons, after relaxing through interactions with phonons, will be located at the minimum of the confining potential at low temperatures. As the temperature increases, the excitonic population starts to gain thermal energy, reaches higher energy states, and spreads over the density of states. This causes an apparent shift of the PL peak to higher energies (blueshift), as observed in Fig. 4d. For even higher temperatures, the thermal energy decomposes a great part of the localized excitons and the shift of the PL peak to lower energies (redshift) prevails, dictated by the thermal dependence of the band gap, which in turn is induced by electron-phonon interaction and lattice thermal expansion.

In order to understand the mechanism of carrier thermal quenching in this system, we fit the experimental data of the integrated PL intensity by using the Arrhenius relation involving two processes [27, 34, 35]:

1 $$ I(T)={I}_o/\left[1+{C}_1 \exp \left(-{E}_1/ kT\right)+{C}_2 \exp \left(-{E}_2/ kT\right)\right] $$

where *T* is the measured temperature, *I*(*T*) is the integrated PL emission intensity, *I*_0_ is a variable parameter, *k* is Boltzmann’s constant, *C*_1_ and *C*_2_ are the ratios of nonradiative to radiative recombination probabilities for the two loss mechanisms, and *E*_1_ and *E*_2_ are the thermal activation energies, and here, the indices, 1 and 2, indicate the constants governing the emission in the low- and high-temperature regimes, respectively. *E*_1_ generally corresponds to the binding energy or detrapping energy of carriers, while *E*_2_ generally corresponds to the energy difference between discrete energy levels or from the discrete energy level to the continuum energy state that is required by the carrier to jump out of the quantum-confined energy state. The best fits yield the activation energies for both QW samples.

**Fig. 5:**
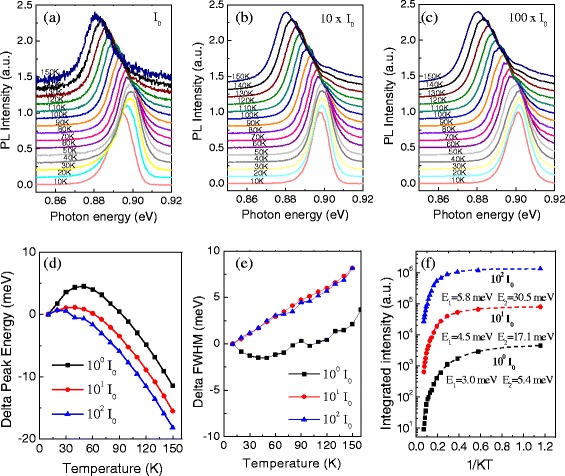
Temperature-dependent PL for the 7-nm QW. The PL spectra as a function of temperature with different excitation intensity of **a** *I*_0_ = 1 W/cm^2^, **b** 10 W/cm^2^, and **c** 100 W/cm^2^. **d** Change of PL peak energy as a function of temperature, **e** change of FWHM of PL spectra as a function of temperature, and **f** integrated PL intensity of PL spectra as a function of temperature

The Arrhenius fits are plotted in Fig. 4e along with the experimental data, and the activation energies generated from the fits are tabulated correspondingly in the plots. Here, we find that the 7-nm QW appears to have small activation energy for both *E*_1_ and *E*_2_, indicating that the localized carrier’s PL quenching are dominant for the 7-nm QW sample under weak excitation condition. In particular, the small activation energy *E*_2_ indicates that it does not correspond to the energy difference between discrete energy levels or from the discrete energy level to the continuum energy state but probably corresponds to the small energy that is required by localized carriers to jump out to enter the free exciton states. This assumption is tested later in Fig. 5f by measuring *E*_2_ with increasing excitation intensity. In comparison, the 15-nm QW has a larger high-temperature activation energy, *E*_2_ = 25.3 meV, indicating carriers are swept out of the QW during the thermal quench process. The different activation energies testify that these two QWs have different thermal quenching mechanisms.

Figure 5a–c further study the temperature-dependent PL for the 7-nm QW at different laser excitation intensities of 1, 10, and 100 W/cm^2^, respectively. Figure 5d shows the behavior of the PL peak energy as a function of temperature for each intensity. When the excitation intensity increases, the maximum blueshift and the temperature at which that maximum blueshift is obtained are changed. For weaker excitation intensities, the maximum blueshift is larger. We have already seen in Fig. 3a that the PL blueshifts with power throughout most of the studied range due to filling of the broadly distributed localized states. The data from Fig. 5 indicate that the temperature-dependent shift follows the same trend and therefore is due to the same mechanism.

The different performance of FWHM as a function of temperature under different excitation intensity is shown as Fig. 5e. According to the previous analysis in Fig. 3, the 7-nm QW emits primarily through the free exciton state for excitation intensities in the range of 10 to 100 W/cm^2^. In Fig. 5e, it can be seen that the FWHM under excitation intensities of 10 and 100 W/cm^2^ follow approximately the same trend. With the temperature increasing, the kinetic energy of the free exciton increases, leading to larger FWHM. However, at the excitation intensity of 1 W/cm^2^, the FWHM first narrows as the temperature increases from 10 to 50 K, because localized excitons occupy a broad distribution of inhomogeneous fluctuations and become thermally excited into the narrower distribution of the free exciton state. After that, the FWHM keeps growing as the temperature increases similar to the high-power situation.

Figure 5f shows the Arrhenius plot at each excitation intensity to investigate the PL quenching behavior. Here, we find, similar to the blueshift, that the thermal quenching characteristics of the 7-nm QW with high-power excitation resembles that of the 15-nm QW under low-power excitation. The activation energy increases from *E*_2_ = 5.4 meV to *E*_2_ = 30.5 meV with increasing excitation laser intensity from 1 to 100 W/cm^2^. As a result, we can conclude, again, that the main channel for recombination under high-power excitation is through the free exciton state for the 7-nm QW.

In order to completely understand the mechanisms underlying the PL observations, we calculate the InGaAs/InAlAs QW band structure using an eight-band k·p model in the Nextnano software based on the detailed structural information obtained from the XRD. The QW thickness is taken with the designed 7 and 15 nm. The material parameters for the calculations are taken from the review article [36] and evaluated at 10 K. To calculate the band profile, we neglected the strain caused by the small mismatch between the In_0.53_Ga_0.47_As QW layer and the In_0.52_Al_0.48_As barrier layer.

**Fig. 6:**
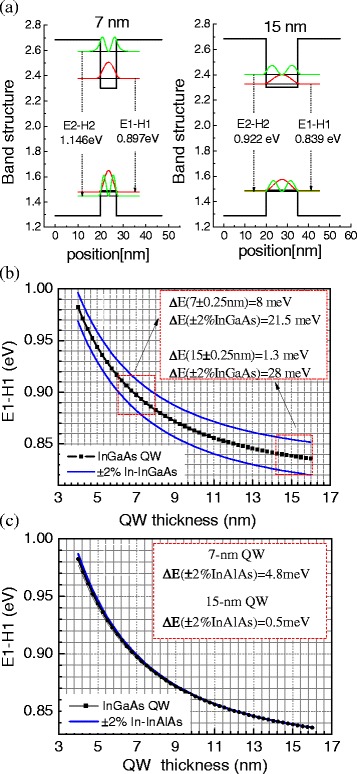
**a** Band profiles of the InGaAs/InAlAs QW with well thickness of 7 and 15 nm, **b** the E1-H1 transition energy as a function of the QW thickness with In composition varied ±2% in the InGaAs QW layer, and **c** the E1-H1 transition energy as a function of the QW thickness with In composition varied ±2% in the InAlAs barrier layers

The band profiles of InGaAs/InAlAs QWs with thicknesses of 7 and 15 nm are computed as shown in Fig. 6a. It can be seen that the calculated E1-H1 emission energies match the experimental results very well. In particular, the calculated E1-H1 transition of 0.897 eV (7-nm QW) at 10 K (Fig. 6a) coincides well with the measured PL peak energy under excitation of 10I_0_ (Fig. 5b). This coincidence also supports the observed turning point of FWHM at ~10I_0_ for both samples (Fig. 3d), suggesting that the ground state is fully filled at ~10I_0_ and broadening of PL peaks at higher excitation densities are caused by filling of excited states. The comparison of these calculations with the PL spectra also confirms that the band structure of InGaAs/InAlAs QW can be easily tailored by adjusting the QW thickness. By decreasing the thickness from 15 to 7 nm, both E1 in the conduction band and H1 in the valence band of the InGaAs QW shift accordingly, leading to an increased E1-H1 emission from 0.839 to 0.897 eV.

In Fig. 6b, c, the calculated E1-H1 emission energies are shown as functions of the QW thickness while incorporating a variation of ±2% in the indium composition of both the InGaAs QW (Fig. 6b) and the InAlAs barrier (Fig. 6c). By examination of these calculations, several conclusions can be extracted. First, In-composition variation in the InGaAs QW has much more effect on E1-H1 transition energy than it does in the InAlAs barrier. For example, for the 7-nm QW, 2% In-composition variation in the InGaAs QW gives an energy change of Δ*E*(±2%InGaAs) = 21.5 meV, while it is only Δ*E*(±2%InAlAs) = 4.8 meV for 2% In-composition variation in the InAlAs barrier. Second, for the same In-composition variation in the InGaAs layer, the thin QW has less change on the E1-H1 transition than the thick QW. The calculation indicates that, for 2% In-composition variation in InGaAs layer, the 7-nm QW has a shift of Δ*E*(±2%InGaAs) = 21.5 meV for the E1-H1 transition in comparison with that of Δ*E*(±2%InGaAs) = 28 meV for the 15-nm QW. Third, for the same amount of thickness fluctuation, the E1-H1 energy of the thin QW changes more than that of the wide QW. The calculated plot shows an energy variation of Δ*E*(7 ± 0.25 nm) = 8 meV for a thickness fluctuation of 0.25 nm (~1 ML) for the 7-nm QW, but it is Δ*E*(15 ± 0.25 nm) = 1.3 meV for the 15-nm QW. Therefore, the composition fluctuations inside the InGaAs layer have a stronger effect on the optical properties for the wide QW, but thickness fluctuations of the InGaAs layer have a stronger effect on the narrow QW. Also, composition fluctuations in the InGaAs affect the wide QWs more than they do the InAlAs barrier [37].

By comparing the calculated curves with the PL results, we speculate that the optical performance of the 7-nm QW is affected more by the interface fluctuation in comparison with the 15-nm QW, although both QW samples may have the same interface roughness or composition fluctuations. In general, there are always interface fluctuations and interface intermixing during the epitaxial growth of compound semiconductor heterostructures. In this case, a better understanding of the role played by the fluctuations of QW thickness, and composition on the optical properties of the InGaAs/InAlAs heterostructures, is highly desirable for optimizing the device performance [38, 39]. Based on our experimental observation and simulations, it is very important to obtain smooth, abrupt interfaces for achieving high-quality QW structures. Depending on the application and desired thickness of the InGaAs/InAlAs structures, we should select a different approach to optimize the growth conditions in order to achieve the best quality QW. In order to get high-quality thick QWs, suppressing the composition drift or composition separation inside the QW will be the best choice. In order to get high-quality narrow QWs, however, more attention should be paid for obtaining smooth, abrupt InGaAs/InAlAs interfaces.

## Conclusions

Interface fluctuation effects have been investigated for the lattice-matched InGaAs/InAlAs single QWs with well widths of 7 and 15 nm. The excitation intensity-dependent PL shows that the 7-nm QW has a large, 15 meV, blueshift of the PL peak energy as the laser excitation intensity increases from 0.01 to 100 W/cm^2^. Temperature-dependent PL shows that the PL peak energy of the 7-nm QW sample displays a blueshift at first and then a redshift with increasing temperature, where the magnitude of the blueshift depends on the laser excitation intensity. The lower the laser excitation intensity, the greater the blueshift. These observations are explained by the localized fluctuations at the InGaAs/InAlAs interface. The experimental and calculated results indicate that the thinner QWs are affected more by interface fluctuations, in particular, by the thickness fluctuation but not the composition fluctuation. This work indicates that it is very important to optimize the interface for achieving high-quality InGaAs/InAlAs QW heterostructures. We should select different approaches to optimize the growth to achieve the best quality QWs based on different thicknesses of the designed InGaAs/InAlAs structures.
